# Metabolic Profiling of the Soft Coral *Erythropodium caribaeorum* (Alcyonacea: Anthothelidae) from the Colombian Caribbean Reveals Different Chemotypes

**DOI:** 10.3390/md18010004

**Published:** 2019-12-18

**Authors:** Sandra L. Molina, Abel M. Forero, Farja I. Ayala, Mónica Puyana, Sven Zea, Leonardo Castellanos, Diego Muñoz, Gonzalo Arboleda, Adrián G. Sandoval-Hernández, Freddy A. Ramos

**Affiliations:** 1 Departamento de Química, Facultad de Ciencias, Universidad Nacional de Colombia Sede Bogotá, Av Cra 30 45-03, 111112 Bogotá, Colombia; sanlimo@gmail.com (S.L.M.); abmforerotu@unal.edu.co (A.M.F.); farjaiayala@gmail.com (F.I.A.); lcastellanosh@unal.edu.co (L.C.); drmunozc@unal.edu.co (D.M.); agsandovalh@unal.edu.co (A.G.S.-H.); 2 Departamento de Ciencias Biológicas y Ambientales, Facultad de Ciencias Naturales e Ingeniería, Universidad Jorge Tadeo Lozano Sede Bogotá, Cra 4 22-6,1, 110010 Bogotá, Colombia; monica.puyana@utadeo.edu.co; 3 Instituto de Estudios en Ciencias del Mar-CECIMAR, Universidad Nacional de Colombia Sede Caribe, 470006 Santa Marta, Colombia; sezeas@unal.edu.co; 4 Facultad de Ciencias, Universidad de Ciencias Aplicadas y Ambientales, 111166 Bogotá, Colombia; 5 Instituto de Genética Humana, Universidad Nacional de Colombia Sede Bogotá, 111112 Bogotá, Colombia; gharboledab@unal.edu.co

**Keywords:** marine natural products, Colombian Caribbean Sea, soft corals, *Erythropodium caribaeorum*, diterpenes, metabolomics, erythrolides, erythrolides W and X, cytotoxicity

## Abstract

The Caribbean soft coral *Erythropodium caribaeorum* is a rich source of erythrolides—chlorinated briarane diterpenoids. These compounds have an ecological role as feeding deterrents, with a wide variation in their composition depending on the location where the sample is collected. In Colombia, this soft coral can be found at different locations in the Caribbean Sea including Santa Marta, Islas del Rosario, and Providencia—three environmentally different coral reef areas in the south and southwest Caribbean Sea. In order to evaluate differences in erythrolide composition, the metabolic profiles of samples from each of these locations were analyzed by HPLC-MS. Principal component analysis showed changes in the diterpene composition according to the sample origin. Diterpenes from samples collected at each location were isolated to describe the three chemotypes. The chemotype from Santa Marta was highly diverse, with the new erythrolides W and X together with eight known erythrolides. The sample from Islas del Rosario showed a low diversity chemotype constituted by high amounts of erythrolide A and B. The chemotype from Providencia showed low chemical diversity with only two main compounds—erythrolide V and R. Evaluation of cytotoxic activity against the human cancer cell lines PC-3, MCF7, and A549 showed erythrolides A and B as the more active compounds with IC_50_ values in the range from 2.45 to 30 μM.

## 1. Introduction

Natural products are a promising source of novel anticancer agents. Over the past 36 years, nearly 63% of the new anticancer drugs introduced to the pharmaceutical market were derived from or inspired by natural products [1]. In 2017, at least 1490 new marine natural products were reported and 149 of them (10%) were isolated from cnidarians solely, making them one of the three main sources of novel natural products in nature [2]. *Erythropodium caribaeorum* (Alcyonacea: Anthothelidae), is an encrusting soft coral with a wide distribution on coral reefs in the Caribbean Sea. This is an interesting organism that produces a large diversity of briarane and eunicellane diterpenes [3,4]. Erythrolides are halogenated briarane diterpenoids well known for their highly functionalized structures and their remarkable biological activity such as anti-inflammatory and cytotoxic potential. Eleutherobins are eunicellane glycosides bound to an *N*-Me urocanic acid residue. Marine diterpenes, in particular, are very interesting due to the fact of their cytotoxic activity, stabilizing microtubules in cancer cells with an activity similar to that of paclitaxel [5]. At least 30 erythrolides and 10 eleutherobin analogs have been identified from samples of *E. caribaeorum* collected in different places around the Caribbean Sea, such as the Bahamas [4], Belize [6], the Virgin Islands [7], Jamaica [8], Tobago [7], and the Dominican Republic [9]. These organisms have showed a wide variation in their metabolic profile. In an early attempt to describe the role of erythrolide production in *E. caribaeorum*, the antifeedant activity from erythrolide B and D, but not from erythrolide A, was described. However, until to now, these kind of studies have not continued with the other isolated erythrolides. Additionally, this soft coral has been cultivated in aquaria with the purpose of producing erythrolides and other diterpenes, overcoming the supply issue that is common for marine natural products [10]. Other soft corals such as *Antillogorgia elisabethae* (Octocorallia: Gorgoniidae) have also shown high chemical variability [11,12,13]. In some cases, chemical variation has been proposed to be related with the associated microbial community [14]; however, this seems not to be the case for *E. caribaeorum* [15]. 

As part of our ongoing efforts to characterize the diversity and pharmacological activities of diterpenes from soft corals [16,17], we studied samples of *E. caribaeorum* from the Colombian Caribbean Sea. Despite reports of this soft coral in various sites, such as Islas del Rosario, Isla de Providencia, Golfo de Urabá, Santa Marta, and Islas San Bernardo [18], no chemical studies have been carried out with this species collected in the abovementioned areas.

Some of the best ways to evaluate chemical diversity in biological samples are metabolomic approaches such as metabolic fingerprinting. This approach has been used to obtain an overview of the metabolic state of an organism and reveal changes in metabolites due to the influence of external factors such as temperature and light, among others [19,20,21]. There are several examples illustrating this application in marine organisms. The metabolomic analysis of soft coral samples from Sanya Bay and Wheizhou Island (China) using ^1^H-NMR spectroscopy showed clear differentiation among the extracts of soft corals growing in those locations. The specific markers that contributed to the discrimination of the samples corresponded to terpenes, sterols, and *N*-containing compounds [22]. In another study, the metabolomic analysis of crude extracts from specimens of the soft coral *Sarcophyton* spp. from the Egyptian Red Sea [23] allowed establishing differences in the metabolic profiles between wild and cultured corals. These differences were evident in terms of cembranoid diterpene content. This study also found higher amounts of those metabolites in wild specimens compared to those cultured [23].

Thus, with the aim of establishing the metabolic variability in *E. caribaeorum* for bioprospecting initiatives, in this work we characterized the diterpene diversity in samples from *E. caribaeorum* collected at three geographically distinct locations of the Colombian Caribbean Sea—Santa Marta, Islas del Rosario, and Providencia Island. We used metabolic profiling tools such as LC-MS, as well as the isolation and characterization of the main compounds of each chemotype. Additionally, we evaluated the cytotoxic activity of the isolated compounds against human cancer cell lines in order to contribute to the pharmacological evaluation of the compounds produced by this soft coral.

## 2. Results and Discussion

### 2.1. MS-Based Metabolic Profiling of E. caribaeorum in the Three Study Locations

**Figure 1:**
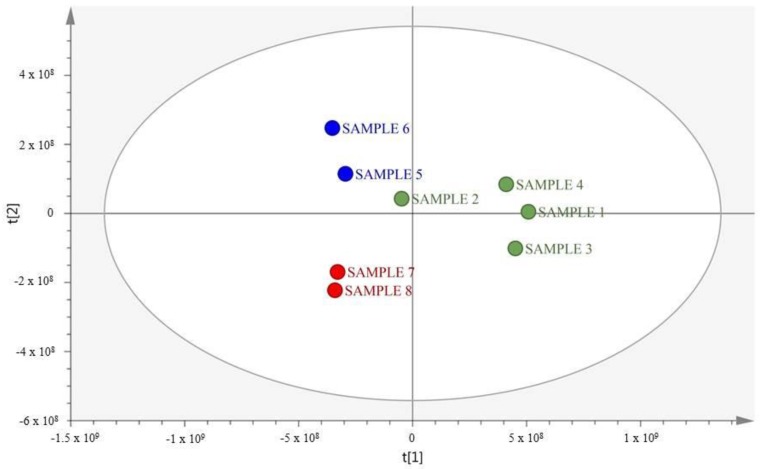
Score plot for the PCA analyses. Green dots represent samples collected at Providencia Island. Blue dots represent samples collected at Santa Marta and red dots represent samples collected at Islas del Rosario (PC1 = 72% and PC2 = 12%; *R*^2^ = 0.844; Q^2^ = 0.71).

For the analysis of the eight samples of *E. caribaeorum* collected at the three different locations of the Colombian Caribbean, HPLC-MS data analyses for pattern recognition were performed by the unsupervised principle component analysis (PCA) method (Figure 1). For PCA, the dimensionality of the input data was defined by the number of samples (variables) and RT-*m/z* pairs (object). In this study, there were eight variables and 24,373 objects. In total, two principal components (PCs) were generated, explaining 84% of the variance.

The PCA analysis of the LC-HRMS data revealed three well-differentiated groups, Group 1—Providencia Island (Samples S1, S2, S3, and S4), Group 2—Santa Marta (Samples S5 and S6), and Group 3—Islas del Rosario (S7 and S8). Sample 2 from Providencia Island was closer to samples from Islas del Rosario. The observed groups (Figure 1) indicate that the metabolic profile of *E. caribaeorum* could be affected either by genetics or by specific environmental conditions at each location. In addition, the initial sample separation showed two trends. The first group, along the first principal component (PC1) in the negative-side grouped samples from Islas del Rosario (samples S7 and S8) and Santa Marta (samples S5 and S6). A second group in the positive side of the PC1 grouped samples from Providencia Island (samples S1–S4). This separation suggests that the metabolic profile of samples collected at the continental coast of Colombia (Islas del Rosario and Santa Marta) was different to that from samples from Providencia Island, a remote island off the Nicaraguan rise.

**Figure 2:**
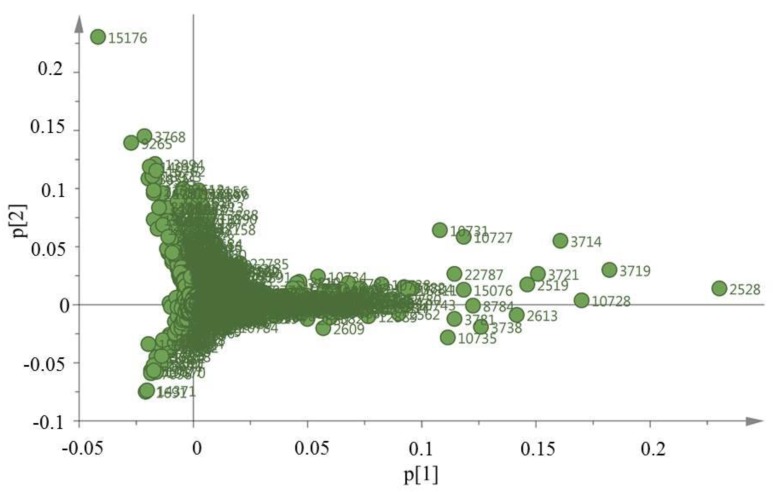
Loadings plot with PCs 1 and 2: here are represented the objects responsible for the separation among the samples.

Loading plots (Figure 2) of PCs 1 and 2 show 24,373 objects described by retention time and *m/z*. Object 2528 could be identified as being responsible for sample separation along PC1. However, no such object assignment could be detected for the observed separation along PC2. The mass spectra for object 2528 indicated signals for a sodium adduct ion at *m/z* 519.1544. Also, its isotopic pattern indicates the presence of a chlorine atom. This evidence suggests a molecular formula C_24_H_29_O_9_Cl, that could correspond to aquarolide A, erythrolide C, erythrolide E, erythrolide R or erythrolide V, previously reported from *E. caribaeorum* [8,9,10]. Other variables of the projection that explained the observed separation among samples had the same *m/z* ions—namely, 519.15—but with slight differences in their retention times (Table S1, Supplementary Materials). This suggests the presence of several of the isobaric compounds mentioned above. In order to identify these and other compounds responsible for the PCA-described chemotypes, the isolation of the main compounds present in the extracts from the three sampling locations was conducted.

### 2.2. Chemical Characterization of E. caribaeorum Chemotypes

**Figure 3:**
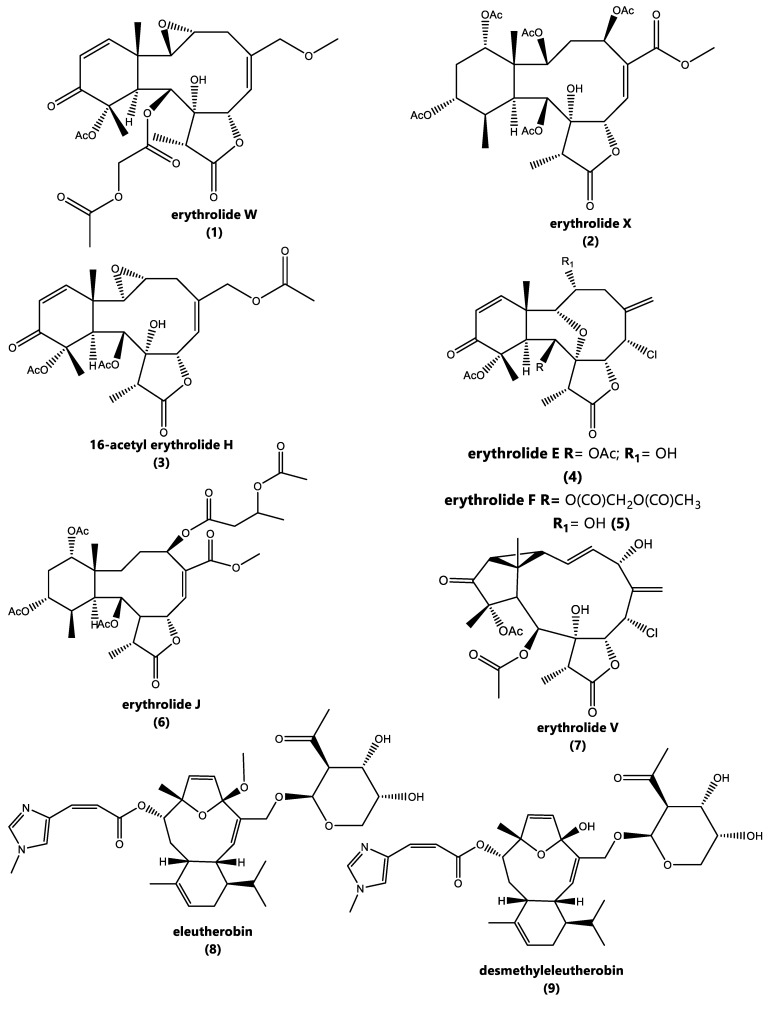

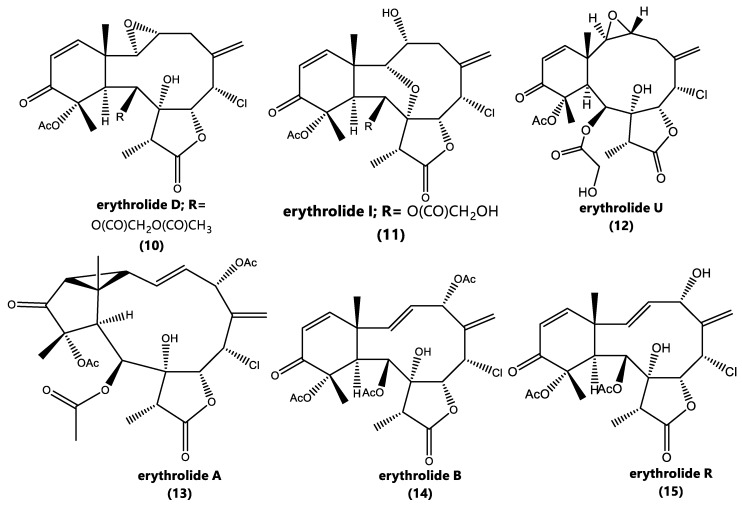
Compounds isolated from three samples (S4, S6, and S8) of *E. caribaeorum* from the Colombian Caribbean.

The crude extracts of samples S4, S6, and S8 were fractionated as mentioned in Section 2.6 to yield sixteen compounds (**1**–**16**) (Figure 3).

Compound **1** (6 mg) was isolated as a white amorphous solid with ${\left[\alpha \right]}_{D}^{23}$ = −25.7 (*c* 0.1, CHCl_3_). Its mass spectrum (HRESIMS) showed a signal for an ion at *m/z* 573.1977 corresponding to its sodium adduct [M + Na]^+^ consistent with the molecular formula C_27_H_34_O_12_ (calcd for C_27_H_34_O_12_Na *m/z* = 573.1948, ∆ = 5 ppm). The ^1^H-NMR (300 MHz, CDCl_3,_ Table 1) showed signals characteristic of erythrolides, with one α,β-unsaturated system in *cis* configuration at δ_H_ 6.67 (1H, d, *J* = 10.2 Hz), 6.00 (1H, d, *J* = 10.2 Hz), and a third proton on a trisubstituted double bond at δ_H_ 5.54 (1H, d, *J* = 10.1 Hz). Two downfield carbinolic protons at δ_H_ 5.79 (1H, bs), 5.14 (1H, d, *J* = 10.1 Hz), and two oxygenated methylenes at δ_H_ 4.64 (2H, bs) and 3.76 (2H, bs). In addition, two aliphatic methines at δ_H_ 3.59 (1H, bs) and 2.53 (1H, q, *J* = 6.5 Hz), two protons characteristic of an epoxide ring at δ_H_ 3.51 (1H, d, *J* = 5.6 Hz) and 2.77 (1H, s) and a signal for a methoxyl group at δ_H_ 3.34 (3H, s), along with two diasterotopic protons for a methylene group at δ_H_ 2.89 (1H, dd, *J*= 16.0, 6.5 Hz) and 2.23 (1H, m) were observed. Finally, five methyls, two of them characteristic of acetyl groups at δ_H_ 2.05 (3H, s) and 2.21 (3H, s), and the other three at δ_H_ 1.48 (3H, s), 1.24 (3H, d, *J* = 6.5 Hz), and 0.94 (3H, s), were observed.

In the APT (attached proton test) experiment (75 MHz, CDCl_3_, Table 1), twenty-seven signals were observed. One of them corresponding to an α,β-unsaturated ketone carbonyl at δ_C_ 194.8, four ester carboxyl resonance signals at δ_C_ 175.8, 170.6, 170.4, and 166.8, two double bonds at δ_C_ 153.6 (CH), and 145.4 (C), 124.1 (CH), and 119.4 (CH), in addition to signals for quaternary carbons bearing oxygen at δ_C_ 81.6 (C) and 81.5 (C), and six carbons bearing oxygen at δ_C_ 77.9 (CH), 76.1 (CH_2_), 70.4 (CH), 63.7 (CH), 61.2 (CH_2_), and 57.2 (CH), the last two characteristic of an epoxide ring. Additionally, one signal at δ_C_ 58.7 (CH_3_) for a methoxyl group, and signals at δ_C_ 43.6 (CH), 41.2 (CH), 41.0 (C), 28.6 (CH_2_), and five methyls at δ_C_ 21.5, 21.3, 20.3, 14.4, and 6.9, were detected.

**Figure 4:**
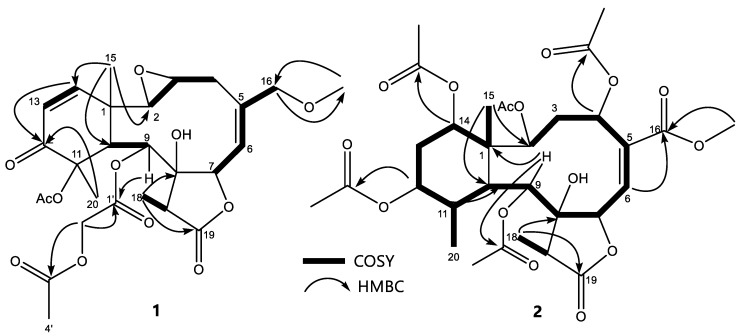
2D planar structure of compounds **1** and **2** and their most important COSY and HMBC correlations.

The 1D NMR data of **1** were similar to those reported for erythrolide H, a previously diacetylated briarane diterpene, isolated from *E. caribaeorum* collected at the Virgin Islands and Jamaica [24]. However, some differences were found, namely, the presence of one methoxyl group (δ_C_ 58.7) and signals for an acetoacetoxy group at δ_C_ 61.2, 170.4, 166.8, and 20.3, a substituent commonly found in other erythrolides [24,25]. Assignation of these substituents was done by the key HMBC correlations between the methoxyl proton (δ_H_ 3.34) and the carbinolic methylene at the 16 position (δ_C_ 76.1) and the correlation from H-9 (δ_H_ 5.79) and the carbonyl of the acetoxyacetyl group (δ_C_ 166.8), to establish the planar structure of **1** as it is presented in Figure 4.

**Figure 5:**
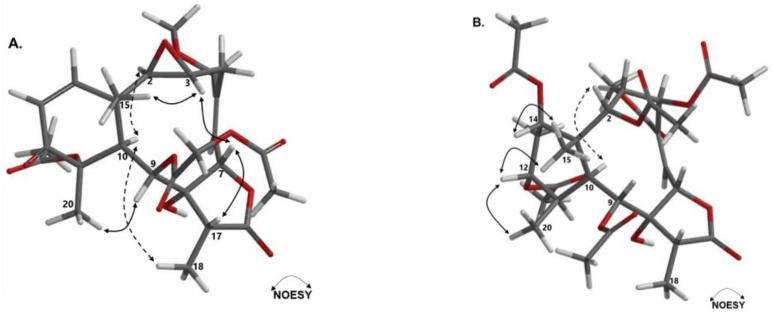
NOESY correlations for compounds **1** (**A**) and **2** (**B**).

The relative stereochemistry was assigned by the interpretation of the NOESY spectrum, (Supplementary Materials, Figure S6). Protons at positions 3, 7, 15, and 17 appear as co-facial by the observed correlations between the CH_3_-15 (δ_H_ 0.94) and the CH-3 proton (δ_H_ 3.51) as well as by the correlation between proton CH-7 (δ_H_ 5.14), CH-3 (δ_H_ 3.51), and CH-17 (δ_H_ 2.53), as is presented in Figure 5 (this figure was generated by a conformer distribution using the software Spartan ’08 with molecular mechanics and MMFF). On the other side, protons at positions 2, 10, and the methyl 18 were also proposed as co-facial by the correlations from the CH-10 proton (δ_H_ 3.59) with two proton signals assigned at CH-2 (δ_H_ 2.77) and CH_3_-18 (δ_H_ 1.24) (Figure 5). In addition, the correlation of protons CH_3_-20 (δ_H_ 1.48) and CH-9 (δ_H_ 5.79), confirms that these protons are co-facial. This proposal is in agreement with structures of previously reported erythrolides [9,10,25]. In addition, the proposed trans configuration for the epoxide ring agrees with the observed multiplicity for proton H-2 (δ_H_ 2.77, bs) and was similar to that reported for other erythrolides with this motif, such as erythrolide T and erythrolide U [9]. This analysis allowed for proposing the structure of compound **1** (Figure 4) as erythrolide W.

Compound **2** (8 mg) was isolated as a white solid with ${\left[\alpha \right]}_{D}^{23}$ = +41.71 (*c* 0.1, CHCl_3_). It showed signals for a [M − H − AcO − Ac]^−^ ion at *m/z* 551.2129 by HRESIMS, consistent with the molecular formula C_27_H_36_O_12_ (calculated for C_27_H_35_O_12_ *m/z* = 551.2124, ∆ = 0.9 ppm). The ^1^H-NMR (400 MHz, CDCl_3_, Table 1) showed signals characteristic of erythrolides, with one proton on a trisubstituted double bond at 6.81 (1H, d, *J* = 10.1 Hz), six hydroxymethine protons at δ_H_ 5.81 (1H, dd, *J* = 11.8, 6.1 Hz), 5.61 (1H, d, *J* = 10.1 Hz), 5.33 (1H, d, *J* = 1.2 Hz), 5.26 (1H, dd, *J* = 12.5, 6.6 Hz), 4.90 (1H, bs), and 4.85 (1H, d, *J* = 8.2 Hz), five of them characteristic of carbon bound to acetyl groups. Two diasterotopic methylenes were observed at δ_H_ 3.00 (1H, m)–2.12 (1H, m) and 2.64 (1H, dd, *J* = 15.2, 7.5 Hz)–2.52 (1H, dd, *J* = 15.2, 6.3 Hz). In addition, there were two aliphatic methines 2.91 (1H, d, *J* = 2.6 Hz) and 2.56 (1H, m), along with one signal of a methoxyl group at δ_H_ 3.84 (3H, s). Finally, seven methyls, five of them characteristic of acetyl groups at δ_H_ 2.24 (3H, s), 2.01 (6H, s), and 1.98 (6H, s), and the three other at δ_H_ 1.30 (3H, d, *J* = 7.1 Hz), 1.28 (3H, d, *J* = 6.4 Hz), and 1.15 (3H, s), were observed.

The APT and HMBC spectra (100 MHz, CDCl_3_, Table 1) showed the presence of seven ester carboxyl resonances at δ_C_ 175.5, 170.8, 170.4, 169.3, 169.2, 168.0, and 167.4. Additionally, the spectra showed two signals for one double bond at δ_C_ 138.3 and 138.0; one signal at δ_C_ 82.8 of a quaternary carbon bound to oxygen; five carbons bearing oxygen at δ_C_ 76.0 (CH), 75.7 (CH), 73.0 (CH), 68.3 (CH), and 67.0 (CH); and one methoxyl at δ_C_ 53.0; two aliphatic methylenes at δ_C_ 41.4 and 37.0. Finally, signals for methines at δ_C_ 45.6 (CH), 43.5 (CH), 32.5 (CH) and seven methyls at δ_C_ 21.6, 21.4, 21.1, 20.1, 14.9, 6.4 were detected. 

The 1D NMR data of **2** was similar to those for erythrolide S, a previously reported diterpene with a peracetylated briarane skeleton, isolated from *E. caribaeorum* collected at Dominica [9]. However, some differences were observed such as the presence of an acetate group at C-4 instead of the sidechain of erythrolide S. The assignation of this acetylation pattern was done by key HMBC correlations between the protons at C-14 (δ_H_ 4.82), C-12 (δ_H_ 5.26), C-9 (δ_H_ 5.30), C-4 (δ_H_ 5.81), and C-2 (δ_H_ 4.90) with the carboxylic carbons at δ_C_ 170.4, δ_C_ 169.3, δ_C_ 169.2, δ_C_ 167.4, and δ_C_ 170.8, respectively, establishing the planar structure of **2** as it is presented in Figure 4.

The relative stereochemistry was assigned by interpretation of the NOESY spectrum (Supplementary Materials Figure S13). Protons at the positions 12, 14, 15, and 20 appear as co-facial by the observed correlations between the methyl CH_3_-15 (δ_H_ 1.15) and the protons CH-12 (δ_H_ 5.26) and CH-14 (δ_H_ 4.85), as well as by the correlation between methyl protons CH_3_-20 (δ_H_ 1.30) and CH-12 (δ_H_ 5.26) (Figure 5B). On the other hand, protons at positions 2 ,9, 10, and 18 were co-facial by the observed correlations from the CH-2 (δ_H_ 4.90) with signals assigned to CH-9 (δ_H_ 5.33) and CH-10 proton (δ_H_ 2.91) (Figure 5B). This proposal is in good agreement with the structures previously reported for other erythrolides [9,10,25]. This analysis allowed for the proposing of the structure of compound **2** as erythrolide X (Figure 4).

However, some differences were observed in the chemical shift values of erythrolide X in comparison with previously reported erythrolide S. In this sense erythrolide X was also submitted to a conformer minimization using the same parameters to erythrolide W. From this, the six-member ring for the 14 (*S*) diastereomer was obtained as a twisted boat conformation in the minimized structure, and was the diastereomer that could explain the observed multiplicity between H-14, H-13ax, and H-13eq in the minimized structure for 14 (*S*). It also could explain the coupling constant and the multiplicity observed between H-14, H-13ax, and H-13eq (d, J = 8.2 Hz). 

Compounds **3–15** (Figure 3) were identified as the known 16-acetyl erythrolide H (**3**), erythrolide E (**4**) [24], erythrolide F (**5**) [24], erythrolide J (**6**) [7], erythrolide V (**7**) [9], eleutherobin (**8**) [26], desmethyleleutherobin (**9**) [26], erythrolide D (**10**) [24], erythrolide I (**11**) [24], erythrolide U (**12**) [9], erythrolide A (**13**) [6], erythrolide B (**14**) [6], erythrolide R (**15**), by comparing their NMR data with that reported in the literature 

With all this information, three chemotypes of *E. caribaeorum* from the Colombian Caribbean Sea were recognized. The Santa Marta chemotype had nine erythrolides, two of them new, together with two eleutherobins. This highly diverse chemotype is similar to that from Dominica and Tobago (Table 3) [9,25]. The Islas del Rosario chemotype had two major compounds erythrolides A and B. The Providencia Island chemotype was characterized by erythrolides V and R. Interestingly, the Islas del Rosario and Providencia Island chemotypes were very similar. They have the same erythrolides skeleton with the hydroxyl at C-4 free in the chemotype from Providencia and acetylated in the chemotype from Islas del Rosario. This Islas del Rosario chemotype is very similar to that previously reported for Belize and the Bahamas Islands (Table 2) [4,27].

Briarane-type diterpenoids represent one of the most common natural product families to be isolated from gorgonian octocorals, with over 700 briarane diterpenoids characterized [28]. The biosynthetic origin of this skeleton was proposed as the result of cyclization between C3 and C8 of a cembrane skeleton, another widely distributed diterpene scaffold in marine gorgonian species, followed by further oxidative processing [29]. These oxidative processes are the responsible of the high diversity of briarane diterpenoids, due to the expression of several CYP450 enzyme-isoforms [30]. Even when there are not studies about erythrolide biosynthesis, erythrolide B (**14**) has been proposed to play a key role in the biosynthetic pathway that drives to the diversity of these compounds in *E. caribaeorum* [9]. Erythrolide B (**14**) along with erythrolides A (**13**), erythrolide R (**15**) and erythrolide V (**7**) are the major metabolites of *E. caribaeorum* in the specimens here examined from Islas del Rosario and Providencia. Erythrolide B (**14**) has been proposed as the starting point for oxidation and rearrangement reactions catalyzed by physical factors such as the UV radiation and enzymatic catalysis [9,31]. These transformations could be induced by environmental perturbations [9]. Thus, is probable that the highly diverse chemotype found at Santa Marta is the response of specific conditions at this location that leads to the mentioned transformations. The chemotypes from Islas del Rosario and Providencia have a low chemical diversity and can be associated to a low environmental pressure at these locations that results in the isolation of the precursor of the erythrolide diversity; however, further experiments should be conducted in order to confirm this hypothesis.

In an effort to integrate the chemical characterization results with those results obtained from the metabolic profiling data presented above, the identified objects from the loading plot that explain the observed variability for the samples from Providencia Island correspond to erythrolides R and V, both with sodium adducts at *m/z* 519.14. Those two compounds were found in high amounts (71.47% for the erythrolide R and 12.07% for the erythrolide V of the normalized peak area in the chromatogram) in the samples from Providencia Island.

Eleutherobins are glycosylated diterpenes different to the erythrolides previously found in *E. caribaeorum*, that are very cytotoxic [32]. Eleutherobins were only found as minor components in the Santa Marta samples (data not shown). Our results show that samples of *E. caribaeorum* from the Colombian Caribbean are a prolific source of erythrolides with their potential applications, yet to be evaluated.

### 2.3. Cytotoxic Activity of Erythrolides

*Erythropodium caribaeorum* has been a rich source of briarane diterpenes with over 30 erythrolide analogues described in the literature. However, their cytotoxicity has not been evaluated so far [33,34,35]. The most potent cytotoxic compounds were erythrolides A and B, with IC_50_ values of 2.45 and 6.46 μM, respectively. Both compounds were isolated from samples from Islas del Rosario. Erythrolides A and B showed partially selective cytotoxicity against the PC3 cancer cell line, whereas erythrolides D, F, R, and U showed partially selective cytotoxicity against the A549 cancer cell line (Table 3). Interestingly, erythrolides R and V (Providencia Island chemotype bearing a free OH in C-5) did not exhibit any cytotoxicity, suggesting the role of the acetyl group at C-5 in the observed activity.

## 3. Materials and Methods 

### 3.1. General

^1^H- and ^13^C-NMR (1D and 2D) spectra were recorded on a Bruker Avance 400 spectrometer (Bruker Biospin Corp., Billerica, MA, USA)) (400 MHz for ^1^H and 100 MHz for ^13^C) and a Bruker Avance 300 spectrometer (300 MHz for ^1^H and 75 MHz for ^13^C-NMR) using CDCl_3_ as solvent. The residual solvent signal was used as an internal standard. High-resolution mass data were collected on an Accurate-Mass quadrupole Time-of-Flight (q-TOF) (Agilent Technologies Inc. Lake Forest, CA, USA) mass spectrometer, electrospray interface (ESI) positive mode, nebulizer 50 (psi), gas flow 10 L/min, and gas temperature 350 °C. Fragmentor 175 V and skimmer 75 V, Vpp 750 V. Metabolite diversity was analyzed with a Shimadzu^®^LCMS-IT-TOF (Shimadzu Corporation, Kyoto, Japan), with an ESI. The MS in the *m/z* range of 100–1000 were analyzed using positive and negative ionization modes with the ESI source at 250 °C and a detected voltage of 2000 V.

Column chromatography (CC) was carried out under vacuum using Merck silica gel 60 G (Merck KgaA, Darmstadt, Germany). Flash chromatography was carried out on Macherey–Nagel silica gel 60 (60–220 mesh) (Macherey–Nagel GmbH & Co, Düren, Germany). The HPLC-ELSD was performed on a Thermo Dionex ultimate 3000 system (ThermoFisher Scientific, Waltham, MA, USA), coupled to an ELSD Sedex 85 (Sedere, Olivet, France) detector with a gain of 10 detector and a temperature of 80 °C for the ELSD detector. Preparative HPLC was performed on a Merck-Hitachi 6000 chromatograph coupled to an UV/VIS L-4250 detector. Optical rotations were measured on a Polartronic E (Schmidt + Haensch polarimeter, Berlin, Germany) in 1 mL × 5 cm cells. All used solvents were HPLC grade.

### 3.2. Animal Material

Samples were collected under Permission No. 4 of 10 February 2010 and Anexo 2, Contrato de Acceso a Recurso Genético No 109, 2014 granted by ANLA (Autoridad Nacional de Licencias Ambientales and Ministerio de Ambiente y Desarrollo Sostenible).

Biological samples for metabolomic analyses were collected at three sites in Providencia Island (SW Caribbean), one in Santa Marta bay, and two sites at Islas del Rosario (near Cartagena) (Table 4). Samples were collected by SCUBA diving, carefully detaching some of the encrusting biomass, leaving at least half of the colony in place. Site, depth, and date of collection were registered. Samples were immediately frozen after collection. Small samples were identified by M. Puyana. Voucher samples were deposited at the Collection of Instituto de Ciencias Naturales ICN of Universidad Nacional, Bogotá, Colombia.

### 3.3. Extraction and Fractionation of E. caribaeorum Samples

Samples of *E. caribaeorum* (Table 1) were freeze-dried and extracted in dichloromethane (DCM)/methanol (1:1, *v/v*) three times at room temperature. Extracts were pooled and concentrated under vacuum at 40 °C to yield a dark green crude extract. For each sample, a portion of the crude extract was resuspended in water and partitioned with DCM to yield two phases. Diterpenes were mainly found in the DCM phase. The DCM phase was further fractionated on a solid-phase extraction cartridge SPE cartridge (HyperSep Thermo Scientific Diol) and eluted with a discontinuous gradient of hexane/ethyl acetate 8:2 (Fraction 1), hexane/ethyl acetate 1:1 (Fraction 2), ethyl acetate 100% (Fraction 3), ethyl acetate/methanol 9:1 (Fraction 4), and methanol 100% (Fraction 5). The five fractions were dried and stored in a freezer at −4 °C until analysis.

### 3.4. LC-MS Analysis

The obtained fractions were analyzed on a Shimadzu^®^ LCMS-IT-TOF with an electrospray interface (ESI). Fractions F1, F2, and F3 were dissolved in methanol (1 mg/mL) and 30 µL of each fraction were injected in a Agilent^®^ Zorbax Eclipse plus column (C18 100 mm × 4.60 mm, 3.5 µm) at 20 °C. Elution was performed at a flow rate of 0.7 mL/min for 40 min, with a decreasing polarity gradient starting with 30% acetonitrile for 5 min, then increasing to 50% acetonitrile in 15 min, followed by a second step gradient up to 100% acetonitrile in 10 min, and finally 100% acetonitrile for 10 minutes, for a total run time of 40 min. The mass detection range was 100–1000 *m/z*, using positive and negative ionization modes with the ESI source at 250 °C and a detected voltage of 2000 V. Peaks from 2 to 40 min in each chromatogram were identified.

### 3.5. Preprocessing of LC-MS Data

The LC-MS data files for each one of the three analyzed fractions were converted from .lcd files to netCDF data using the ACD/MS Manager program ver 12.0 (ACD-Labs, Toronto, ON, Canada). Data were then imported into mzMine 2 [36] for preprocessing steps. Data preprocessing included noise filtering, peak detection, deisotoping, alignment, and gap filling [37]. Noise filtering was accomplished by the exact mass detector with a noise level value (intensity) of 4.85 × 10^4^ counts/s (cps) and set appropriately by comparing with a methanol blank. Peaks with an intensity below this level were not recognized for further steps, therefore they were not included in the mass list. The chromatogram builder was set at 0.03 min, while the minimum height required for chromatogram inclusion was 4.85 × 10^4^ cps and *m/z* tolerance of 5 ppm. Chromatogram deconvolution was carried out by the Staviszky-Golay algorithm for the peaks in each one of the built chromatograms. The most intense isotope was chosen to be representative of the parent molecule. Deisotoping was carried out with an *m/z* tolerance of 10 ppm and a retention time tolerance of 0.01 min. The RANSAC (Random sample consensus) alignment was selected as the most suitable alignment method. For gap filling we used the same RT methods and *m/z* range gap filler with an *m/z* tolerance of 5 ppm. Aligned peaks were exported as a comma separated value (.CSV) file. For the statistical analyses, data obtained for each fraction were merged in order to obtain one dataset per sample. In this step, the area under the curve for each signal obtained in the three fractions of a given samples was added. Thus, a single matrix was obtained for the eight *E. caribaeorum* samples. A multivariate statistical analysis was performed with the SIMCA^®^ version 14.1 (Umetrics, Umea, Sweden) software, using the centering scaling method.

### 3.6. Isolation and Identification of Erythrolides and Eleutherobins

Sample 4 (Providencia), sample 6 (Santa Marta), and sample 8 (Islas del Rosario) were selected for isolating compounds. Erythrolides and eleutherobins were isolated using open column chromatography (silica gel 60, 0.063–0.200 mm, Merck) followed by preparative HPLC. The DCM phase of Santa Marta (sample 6) was eluted with a discontinuous gradient of hexane/ethyl acetate, ethyl acetate/methanol and methanol 100%, to yield nine fractions (FS1–FS9). Fraction FS6 (300 mg) was separated by preparative HPLC using a Hibar^®^ LiChrospher RP-18 (250 mm × 25 mm i.d; 7 µm) column (Merck, Darmstadt, Germany), eluting with acetonitrile/water isocratic (40:60) system at a flow rate of 3 mL min^−1^, detecting at 215 nm, to obtain compounds **1** (6 mg), **3** (32 mg), **4** (16 mg), **5** (12 mg), **6** (33 mg), and **7** (3 mg). The separation of fraction FS9 was achieved using a LiChrospher-CN (125 mm × 4 mm i.d; 5 µm) column, eluting with hexane/ethyl acetate (43:57) system at 0.7 mL min^−1^, detecting at 290 nm. This fraction yielded compounds **8** (2 mg) and **9** (1 mg). Fraction FS5 (280 mg) was purified using preparative HPLC in a THERMO SCIENTIFIC (ELSD-UV detector) equipment, with a Kromasil C8 column (10 × 250 mm, 5 mm i.d.), eluting with a multistep gradient of acetonitrile (A) and water (B) as follows: a linear gradient from 40% A up to 50% A in 12 min followed by 50% A for 8 min; a second step linear gradient up to 65% A in 3 min, followed by a third gradient up to 100% A in 2 min; and finally 100% A for 17 min. The flow rate was 2 mL/min for a total run time of 37 min, using UV detection at 254 nm and an ELSD detection at 80 °C and gain value of 8. This fractionation scheme yielded compounds **2** (9 mg), **10** (14 mg), **11** (8 mg), and **12** (4 mg)**.**

The fraction soluble in DCM from Islas del Rosario (sample 8) was fractionated by silica gel 60 column chromatography using a discontinuous gradient solvent from hexane 100% to ethyl acetate 100%, to yield eight fractions FR1–FR8. Fraction R8 (250 mg) was fractionated by preparative HPLC using a Kromasil C18 column (10 × 250 mm, 50 µm), eluting with a gradient of acetonitrile (A) and water (B) as follows, and isocratic (40:60) run for 17 min, followed by a linear increase to 100% A in 15 min and 5 min at 100% A with a flow rate of 2 mL/min. Compounds were detected at a wavelength of 254 nm and an ELSD detection at 80 °C and gain value of 8. In this manner, compounds **13** (16 mg) and **14** (40 mg) were obtained Samples 1, 3, and 4 from Providencia Island, pooled (250 mg), and then fractionated using the previously described scheme to yield compounds **7** (38 mg) and **15** (23 mg).

### 3.7. Cytotoxicity Assay

Cancer cell lines were purchased from ATCC and grown following ATCC’s recommendations. Cell lines A549 (human lung cancer), MCF7 (human breast adenocarcinoma), and PC3 (human prostate cancer) were cultured in Dulbecco’s modified Eagle’s medium (DMEM) (Lonza, Rockland, ME, USA) supplemented with 10% fetal bovine serum (FBS, Gibco, ThermoFisher Scientific, Waltham, MA, USA) and 1% penicillin–streptomycin (Lonza, Rockland, ME, USA) in a humidified atmosphere at 37 °C in 5% CO_2_.

Cytotoxicity was evaluated using the 3-(4,5-dimethyl-2-thiazolyl)-2,5-diphenyl tetrazolium bromide (MTT) assay [38]. Briefly, cells were seeded in 96 well plates (1 × 10^4^ cell/well) and allowed to settle for 24 h. Cells were then treated with different concentrations (100, 30, 10, 1, and 0.1 µg/mL) of the isolated erythrolides (compounds **1**–**7**, **10**–**15**) dissolved in ultrapure dimethyl sulfoxide (DMSO; Sigma–Aldrich, San Luis, Mo, USA) at 0.1 mg/mL. After 48 h, an MTT (ThermoFisher Scientific, Waltham, Ma, USA) solution (10 μL of a 5 mg/mL stock) in PBS was added to each well and incubated for 4 h at 37 °C in 5% CO_2_. Finally, the supernatant was removed and 100 µL of saline lysis buffer was added and absorbance was measured in a Tecan Sunrise Eliza-Reader (Hombrechtikon, Switzerland) at 595 nm.

## 4. Conclusions

Three different chemotypes of the soft coral *Erythropodium caribaeorum* were described based on their diterpene composition using LCMS-based metabolic profiling. The highly diverse chemotype from Santa Marta yielded two new diterpenoids—namely, erythrolides W and X—that were described based on their 1D and 2D NMR data. Chemotypes from Islas del Rosario and Providencia Island were much simpler. The chemotype from Islas del Rosario was characterized by the presence of erythrolides A and B, while the chemotype from Providencia Island was characterized by erythrolides R and V, compounds closely related to the structures of erythrolides A and B. The cytotoxicity evaluation of the isolated erythrolides showed that erythrolides A and B were the most active and promising among all the tested compounds.

## Figures and Tables

**Table 1:** 1D NMR data of (**1**) in CDCl_3_- (^1^H-NMR at 300 MHz; ^13^C-NMR at 75 MHz) and 1D NMR data of (**2**) in CDCl_3_- (^1^H-NMR at 400 MHz; ^13^C-NMR at 100 MHz).

N°	Compound 1		Compound 2	
	δ_H_ integr, mult, (J in Hz)	δ_C_ mult.	δ_H_ integr, mult, (J in Hz)	δ_C_ mult.
1		41.0, C		45.6, C
2	2.77, 1H, s	63.7, CH	4.90, 1H, bs	76.0, CH
3a	3.51, 1H, d, (5.6)	57.2, CH	3.00. 1H, m	37.0, CH_2_
3b			2.12, 1H, m	
4a	2.89, 1H, dd, (16.0, 6.5)	28.6, CH_2_	5.81, 1H, dd, (11.8, 6.1)	68.3, CH
4b	2.23, 1H, m			
5		145.4, C		138.3, C
6	5.54, 1H, d, (10.1)	119.4, CH	6.81, 1H, d, (10.1)	138.0, CH
7	5.14, 1H, d, (10.1)	77.9, CH	5.61, 1H, d, (10.1)	76.0, CH
8		81.5, C		82.8, C
9	5.79, 1H, bs	70.4, CH	5.33, 1H, d, (1.2)	75.7, CH
10	3.59, 1H, bs	41.2, CH	2.91, 1H, d, (2.6)	32.5, CH
11		81.6, C	2.56, 1H, m	43.5, CH
12		194.8, C	5.26, 1H, dd, (12.5, 6.6)	67.0, CH
13a	6.00, 1H, d, (10.2)	124.1, CH	2.64, 1H, dd, (15.2, 7.5)	41.4, CH_2_
13b			2.52, 1H, dd, (15.2, 6.3)	
14	6.67, 1H, d, (10.2)	153.6, CH	4.85, 1H, d, (8.2)	73.0, CH
15	0.94, 3H, s	14.4, CH_3_	1.15, 3H, s	14.9, CH_3_
16	3.76, 2H, bs	76.1, CH_2_		168.0, C
17	2.53, 1H, q, (6.5)	43.6, CH	2.56, 1H, m	43.5, CH
18	1.24, 3H, d, (6.5)	6.9, CH_3_	1.28, 3H, d, (6.4)	6.4, CH_3_
19		175.8, C		175.5, C
20	1.48, 3H, s	21.3, CH_3_	1.30, 3H, d, (7.1)	20.1, CH_3_
1’		166.8, C		
2’	4.64, 2H, bs	61.2, CH_2_		
3’		170.4, C		
4’	2.21, 3H, s	20.3, CH_3_		
AcO-2				170.8, C
			1.99, 3H, s	20.1, CH_3_
AcO-4				169.9, C
			2.01, 3H, s	21.4, CH_3_
AcO-9				169.2, C
			2.24, 3H, s	21.6, CH_3_
AcO-11		170.6, C		
	2.05, 3H, s	21.5, CH_3_		
AcO-12				170.3, C
			1.95, 3H, s	20.7, CH_3_
AcO-14				170.4, C
			1.98, 3H, s	21.1, CH_3_
OMe-16	3.34, 3H, s	58.7, CH_3_	3.84, 3H, s	53.0, CH_3_
				

**Table 2:** Metabolic comparison of *E. caribaeorum* from different locations in the Caribbean Sea. The gray color indicates the presence of the related compound in the specific location.

	Ery A	Ery B	Ery C	Ery D	Ery E	Ery F	Ery G	Ery H	Ery I	Ery J	Ery K	Ery L	Ery M	Ery N	Ery O	Ery P	Ery Q	Ery R	Ery S	Ery T	Ery U	Ery V	Ery W	Ery X
**Jamaica** [8,24]																								
**Belize** [6]																								
**Bahamas** [4]																								
**Tobago** [25,7]																								
**Dominica** [9]																								
**Santa Marta**																								
**Islas del Rosario**																								
**Providencia**																								

**Table 3:** Cytotoxicity of the different erythrolides isolated in this study against the cancer cell lines A549 (human lung cancer), MCF7 (human breast adenocarcinoma), and PC3 (human prostate cancer). Data are presented as the mean ± SE IC_50_ (µM) for the identified chemotypes**.**

ID	IC_50_ A549 (µM)	IC_50_ MCF7 (µM)	IC_50_ PC3 (µM)
erythrolide A	18.41 ± 3.15	6.77 ± 0.98	2.45 ± 0.38
erythrolide B	27.09 ± 4.26	15.21 ± 2.31	6.46 ± 0.96
erythrolide D	2.58 ± 0.72	42.45 ± 4.34	60.00 ± 5.55
erythrolide E	148.22 ± 12.27	73.87 ± 5.03	189.11 ± 14.93
erythrolide F	46.49 ± 7.93	116.44 ± 12.97	>120
erythrolide I	>120	>120	>120
erythrolide J	37.93 ± 3.91	56.06 ± 3.88	42.49 ± 3.53
erythrolide R	75.28 ± 18.31	>120	>120
erythrolide U	36.65 ± 5.84	124.79 ± 15.01	>120
erythrolide V	101.72 ± 20.72	>120	>120
erythrolide W	>120	>120	>120
erythrolide X	>120	>120	>120
16-acetyl erythrolide H	>120	113.11 ± 16.04	>120

**Table 4:** Site, depth, and date of the collection of the samples of *E. caribaeorum* used in this study.

# SAMPLE	LOCATION	DEPTH (m)	DATE	VOUCHER NUMBER
1	Providencia Island (Santa Catalina Cay)	6	16 July 2012	CO 0109
2	Providencia Island (Lawrence Reef)	1.5	28 February 2013	CO 0110
3	Providencia Island (Lawrence Reef)	1.5	28 February 2013	CO 0111
4	Providencia Island (Convento)	7.5	16 July 2012	CO 0112
5	Santa Marta (Punta Venado)	4.5	10 December 2011	CO 0113
6	Santa Marta (Punta Venado)	6	06 September 2010	CO 0114
7	Islas del Rosario (Botellas, Isla Grande)	4.5	20 September 2012	CO 0115
8	Islas del Rosario (Pavitos)	17	22 September 2012	CO 0116

## Supplementary Materials

The following are available online at https://www.mdpi.com/1660-3397/18/1/4/s1, Table S1: Variables responsible for the separation among samples with the same *m/z* ions and similar retention times, Figure S1: 1H-NMR at 300 MHz spectra of erythrolide W in CDCl_3_, Figure S2: APT-NMR at 75 MHz spectra of erythrolide W in CDCl_3_, Figure S3: COSY spectra of erythrolide W in CDCl_3_, Figure S4: HSQC spectra of erythrolide W in CDCl_3_, Figure S5: HMBC spectra of erythrolide W in CDCl_3_, Figure S6: NOESY spectra of erythrolide X in CDCl_3_, Figure S7: 1H-NMR at 400 MHz spectra of erythrolide X in CDCl_3_, Figure S8: APT spectra at 100 MHz of erythrolide X in CDCl_3_, Figure S9: COSY spectra of erythrolide X in CDCl_3_, Figure S10: HMBC spectra of erythrolide X in CDCl_3_, Figure S11: HSQC spectra of erythrolide X in CDCl_3_, Figure S12: HRMS spectra of erythrolide X, Figure S13: NOESY spectra of erythrolide X in CDCl_3_.
