# Supplementary material for: Metabolic Profiling of the Soft Coral Erythropodium caribaeorum (Alcyonacea: Anthothelidae) from the Colombian Caribbean Reveals Different Chemotypes
Source: Mar Drugs. 2019 Dec 18;18(1):4. doi: 10.3390/md18010004 (PMC7024379; doi:10.3390/md18010004)
Supplement: Supplementary file 1 [file marinedrugs-18-00004-s001.pdf]

Metabolic profiling of the soft coral *Erythropodium caribaeorum* (Alcyonacea: Anthothelidae) from the Colombian Caribbean reveals different chemotypes (SUPPORTING INFORMATION)

Sandra L. Molina[a], Abel M. Forero[a], Farja I. Ayala[a], Mónica Puyana[b], Sven Zea[c], Leonardo Castellanos[a], Diego Muñoz[a,d,e], Gonzalo Arboleda[d], Adrián G. Sandoval-Hernández[a,d] Freddy A. Ramos[a*]

[a]Universidad Nacional de Colombia - Sede Bogotá - Facultad de Ciencias - Departamento de Química, Av Cra 30 45-03 Bogotá, Colombia
[b]Universidad Jorge Tadeo Lozano - Sede Bogotá - Facultad de Ciencias Naturales e Ingeniería – Departamento de Ciencias Biológicas y Ambientales, Cra 4 22-61 Bogotá, Colombia
[c]Universidad Nacional de Colombia - Sede Caribe - Instituto de Estudios en Ciencias del Mar-CECIMAR - Santa Marta, Colombia
[d]Universidad Nacional de Colombia - Sede Bogotá - Instituto de Genética Humana, Bogotá, Colombia
[e]Facultad de Ciencias, Universidad de Ciencias Aplicadas y Ambientales, Bogotá, Colombia.
Received: date; Accepted: date; Published: date
*faramosr@unal.edu.co

Table S1. Variables responsible of the separation among samples with the same m/z ions, and similar retention times.

Object	*m/z*	Retention time (minutes)
**2519**	519.15	8.16
**2528**	519.15	7.63
**2613**	519.11	8.30
**3714**	519.15	8.23
**3719**	519.18	8.25
**3721**	519.10	8.30
**3738**	519.15	7.70
**3781**	519.15	5.64
**8784**	519.15	6.38
**10727**	519.15	8.20
**10728**	519.14	6.66
**10731**	519.12	8.22
**10735**	519.15	6.64
**15076**	519.15	8.26
**22787**	519.17	8.25

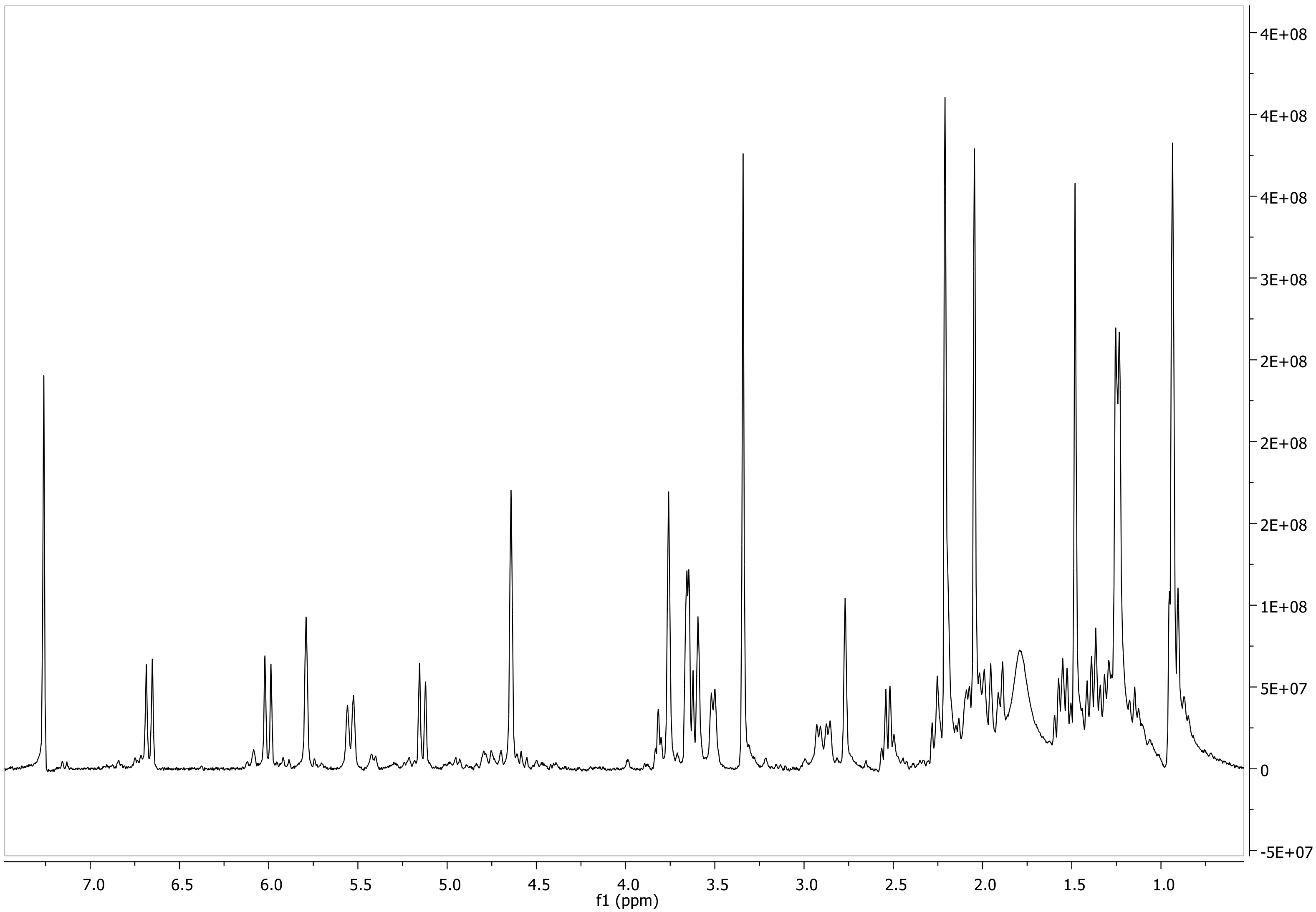

Figure 1S. ^{1}H-NMR at 300 MHz spectra of erythrolide W in $CDCl_3$.

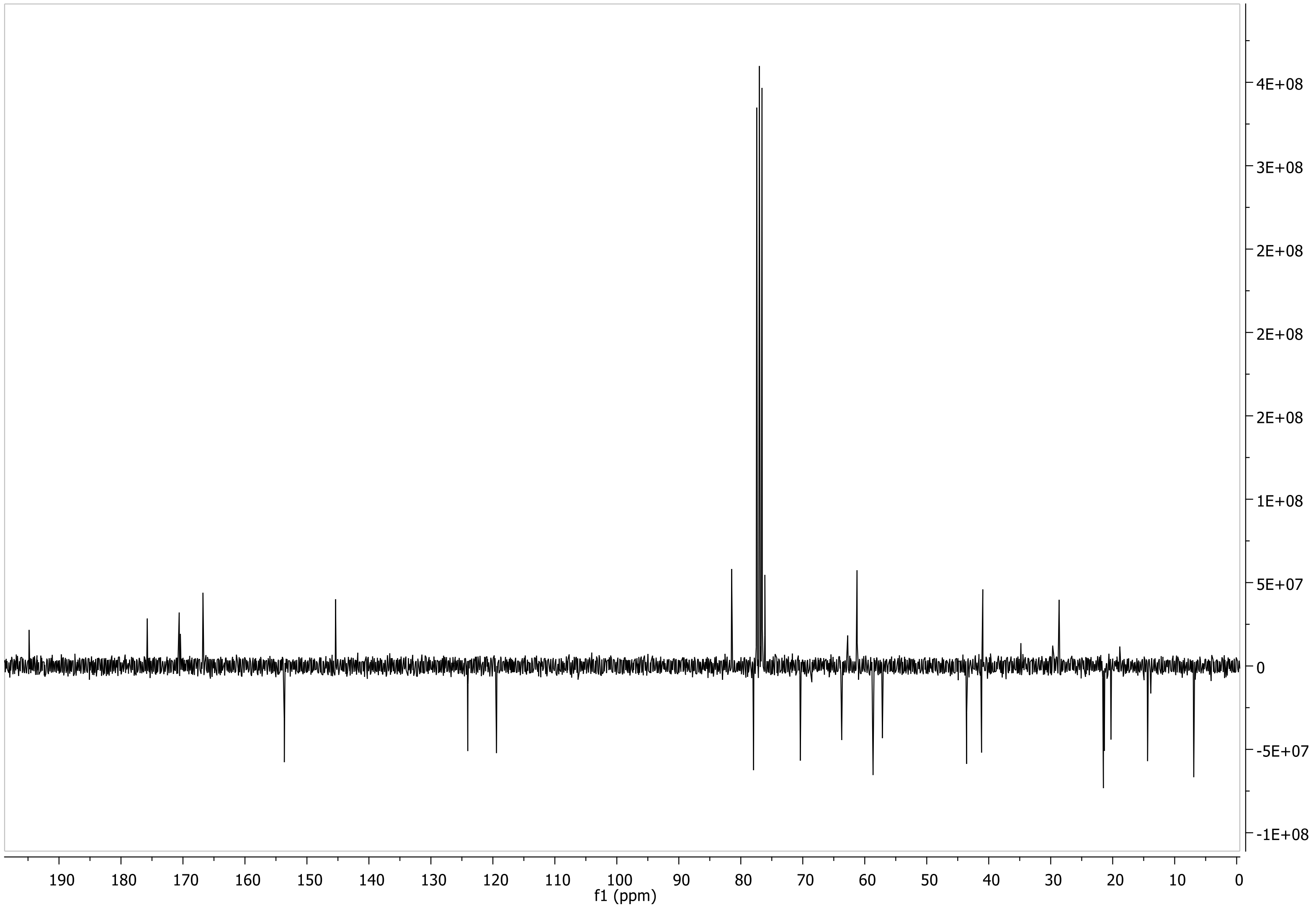

Figure 2S. APT-NMR at 75 MHz spectra of erythrolide W in $CDCl_3$.

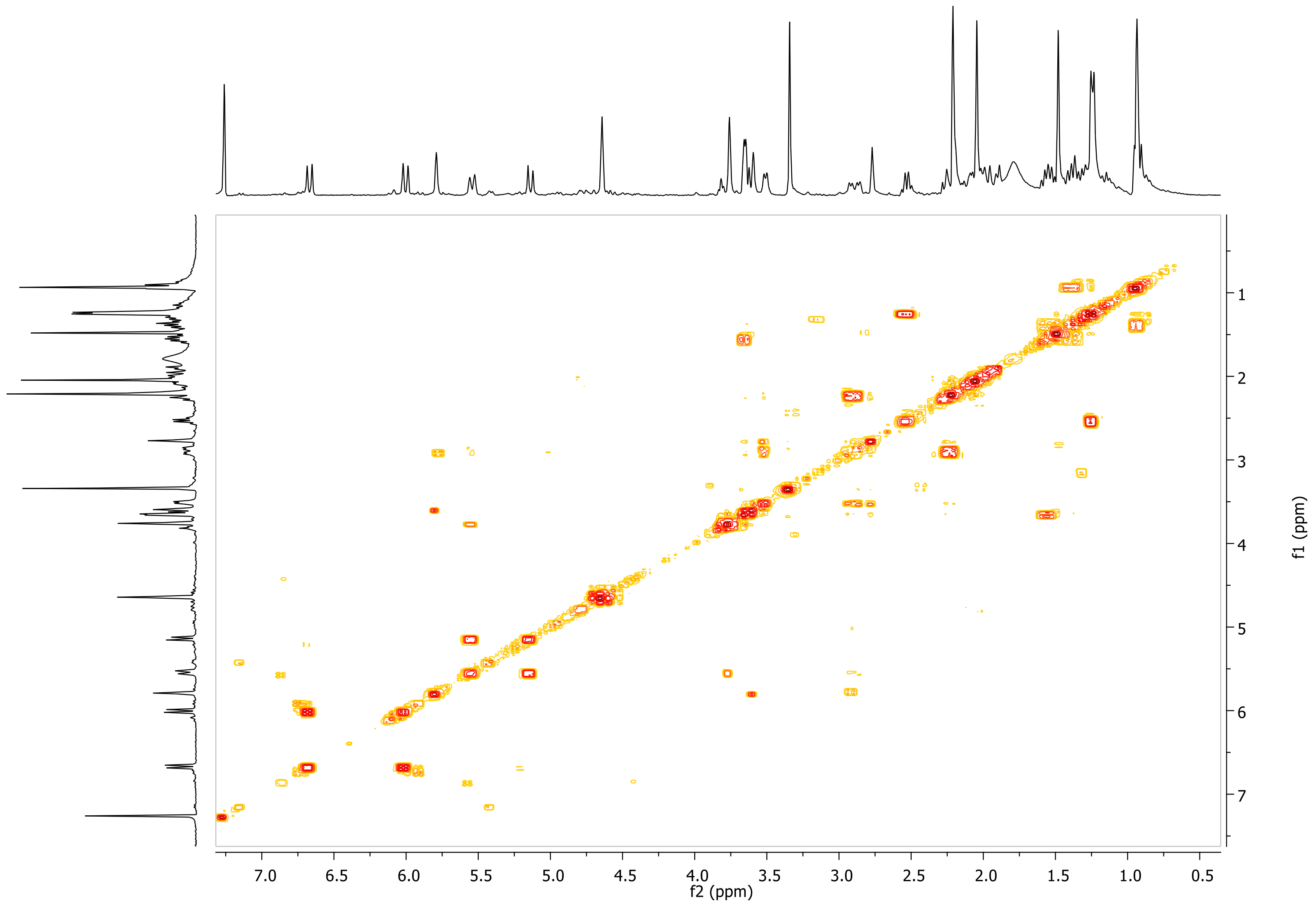

Figure 3S. COSY spectra of erythrolide W in $CDCl_3$.

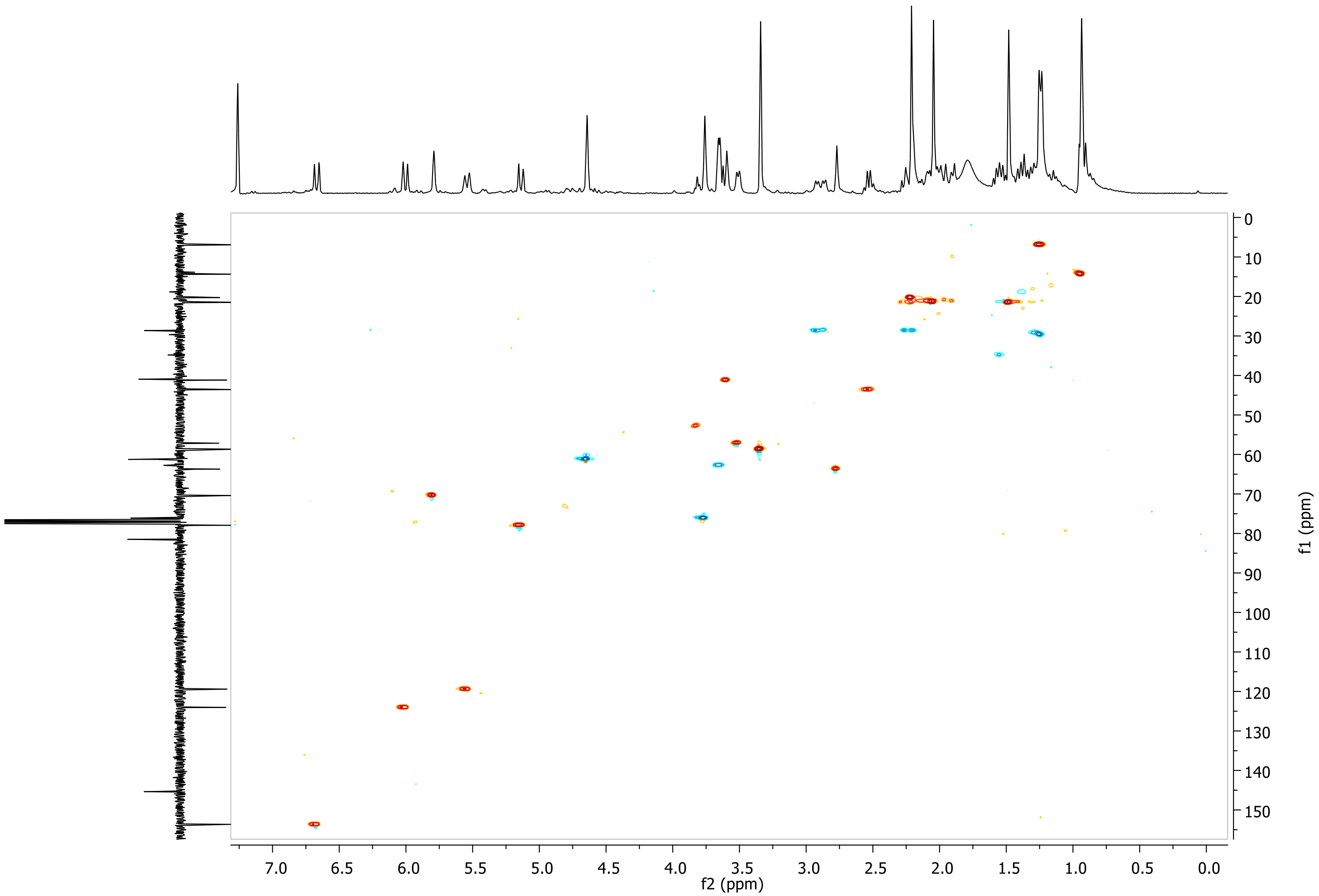

Figure 4S. HSQC spectra of erythrolide W in $CDCl_3$.

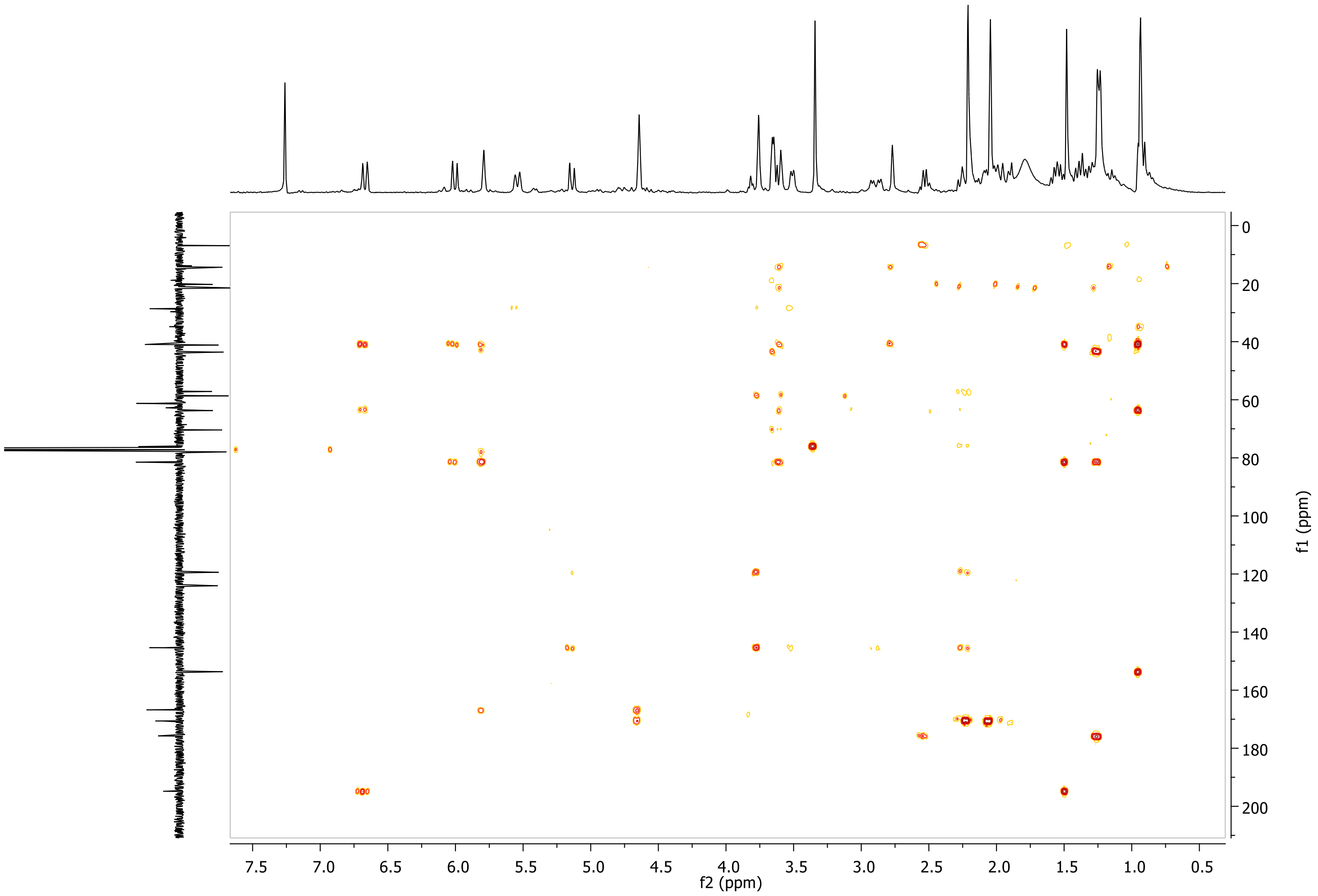

Figure 5S. HMBC spectra of erythrolide W in $CDCl_3$.

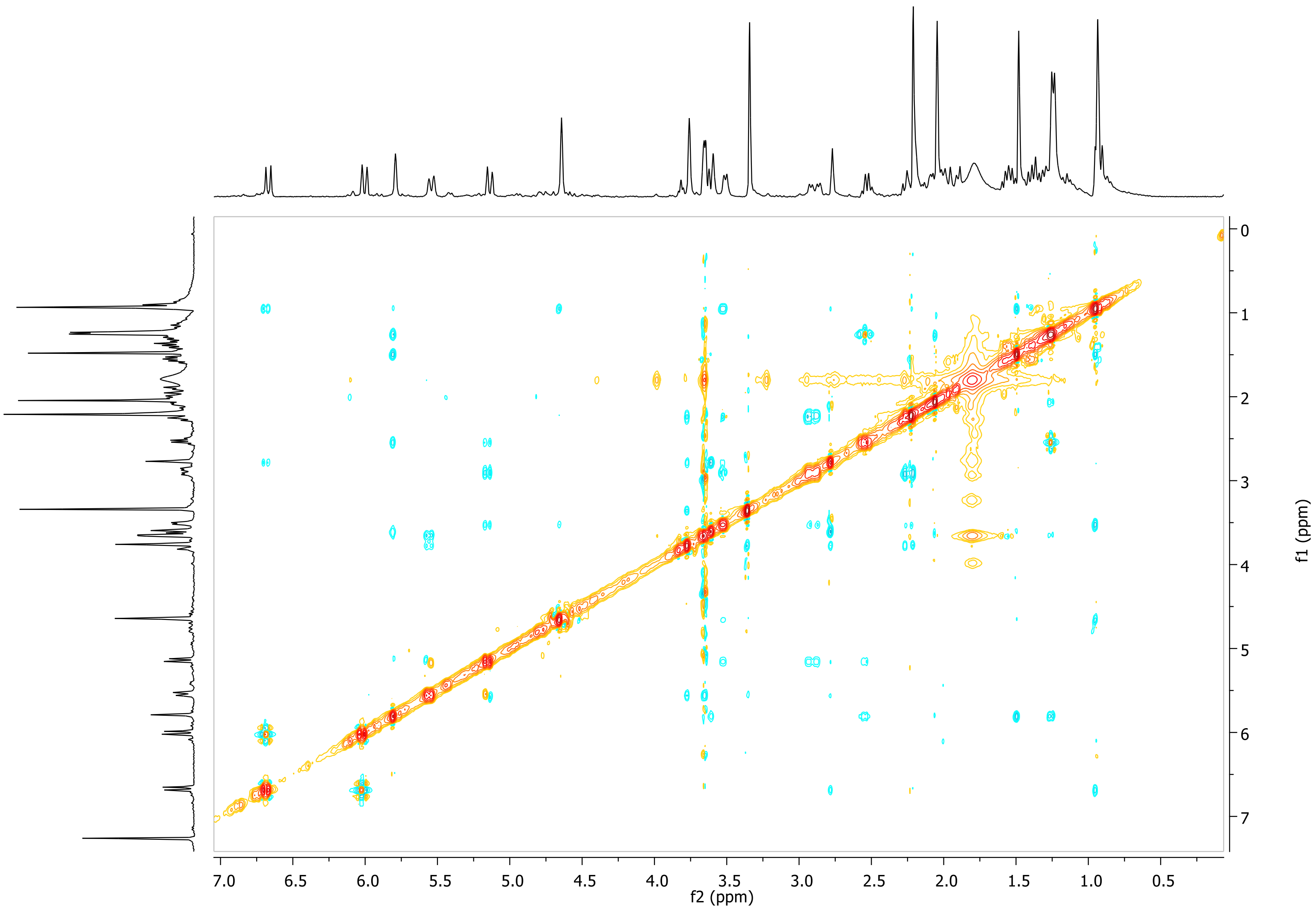

Figure 6S. NOESY spectra of erythrolide X in $CDCl_3$.

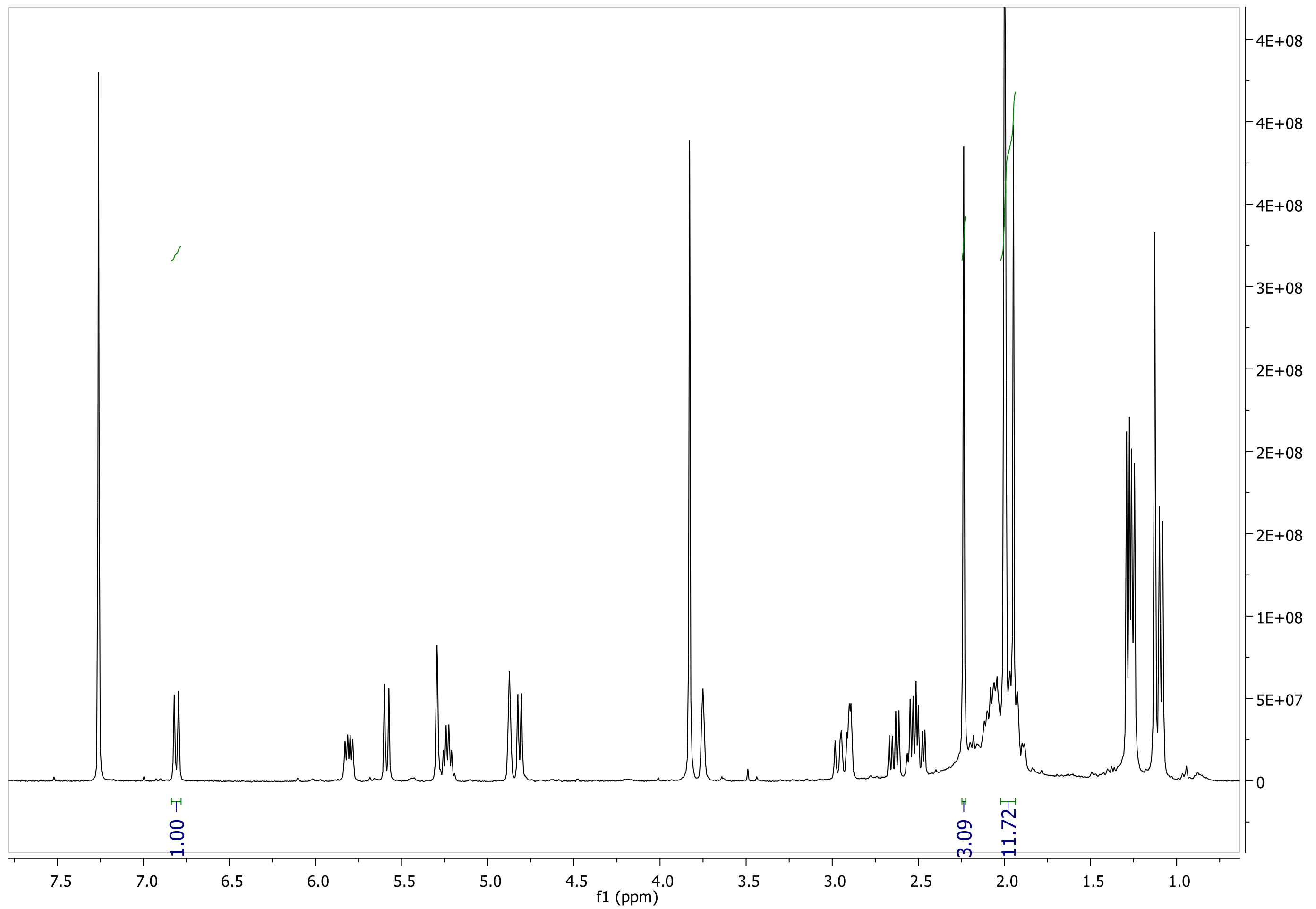

Figure 7S. ^{1}H-NMR at 400 MHz spectra of erythrolide X in $CDCl_3$.

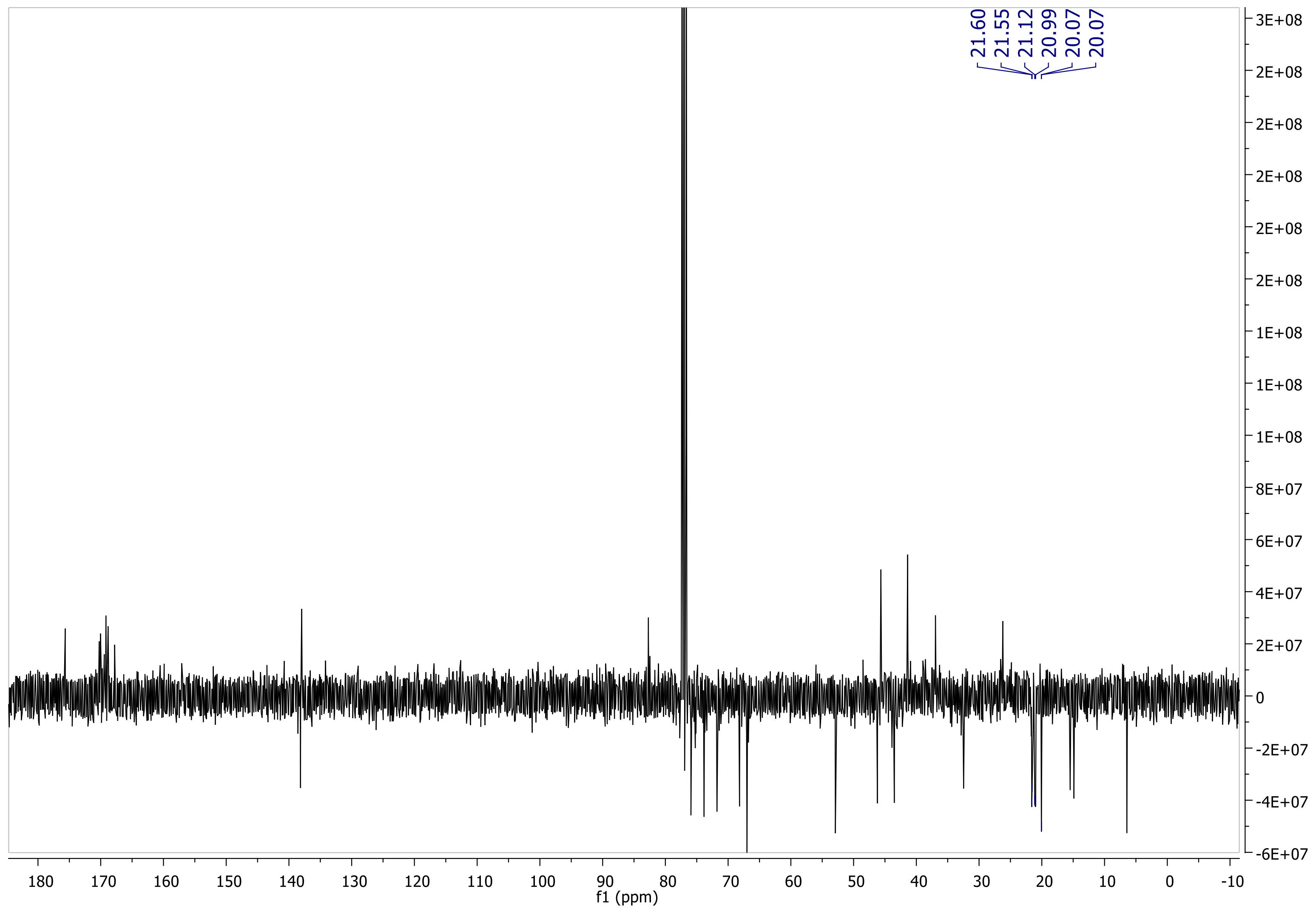

Figure 8S. APT spectra at 100 MHz of erythrolide X in $CDCl_3$.

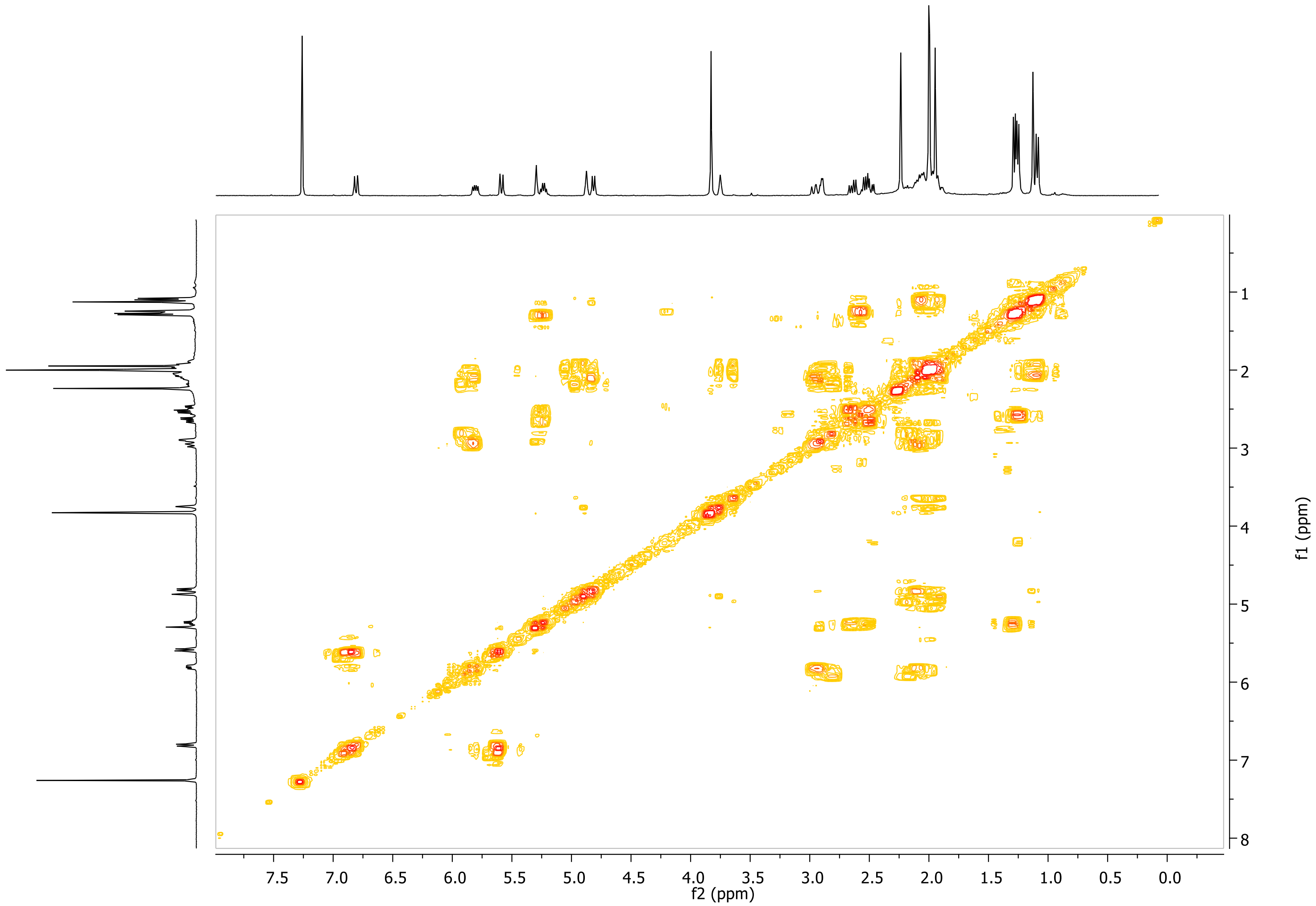

Figure 9S. COSY spectra of erytrholide X in $CDCl_3$.

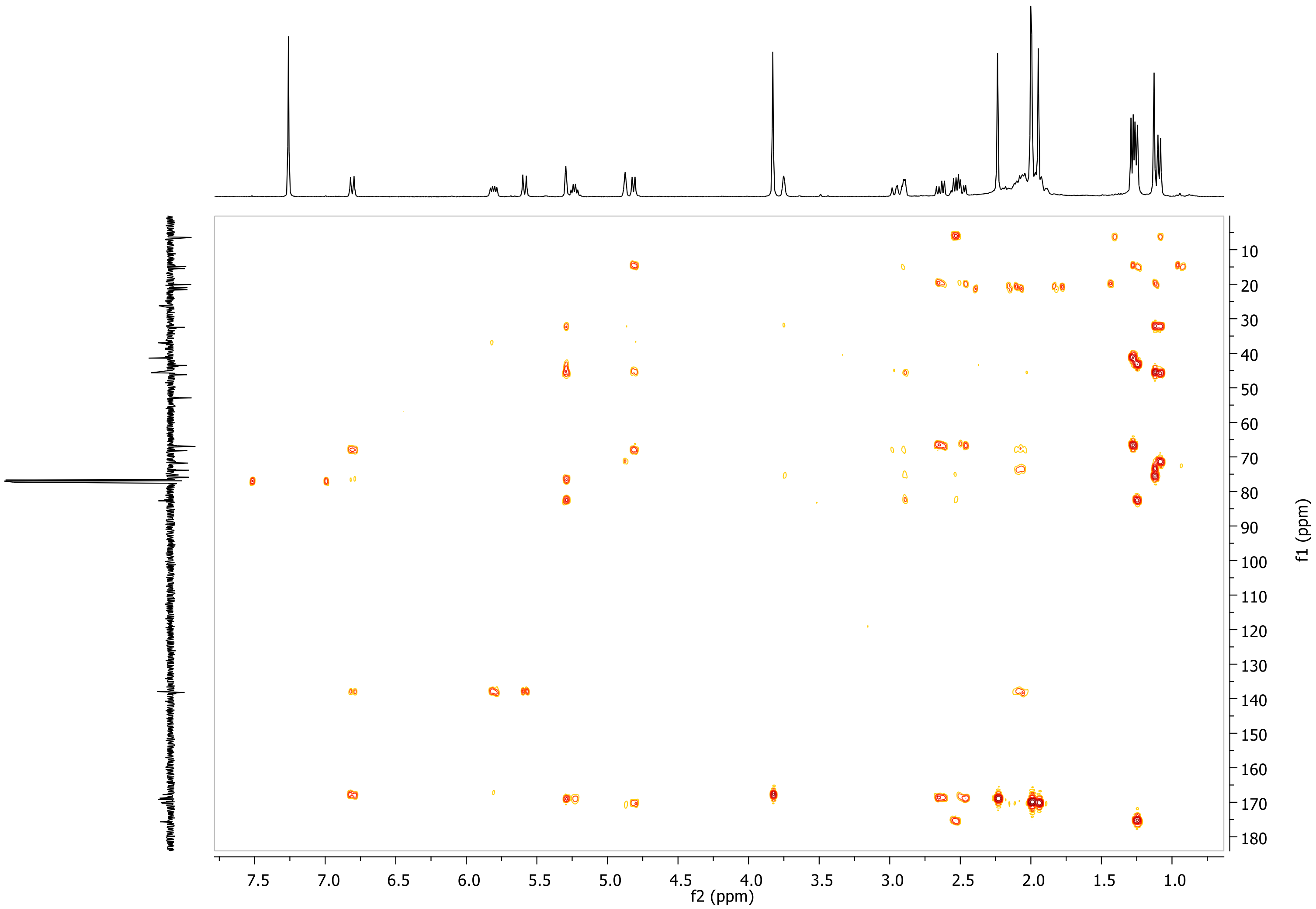

Figure 10S. HMBC spectra of erytrholide X in $CDCl_3$

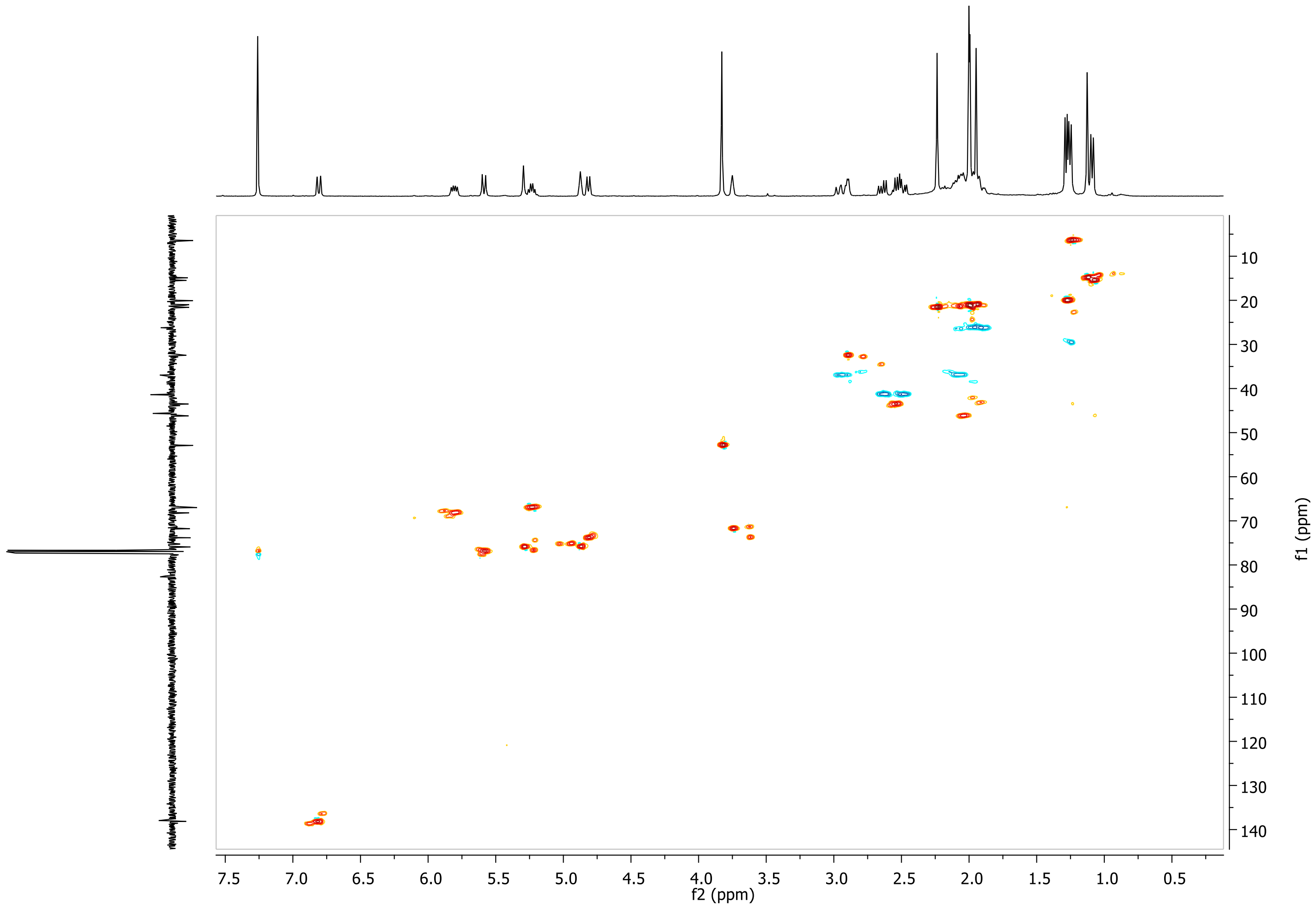

Figure 11S. HSQC spectra of erytrholide X in $CDCl_3$

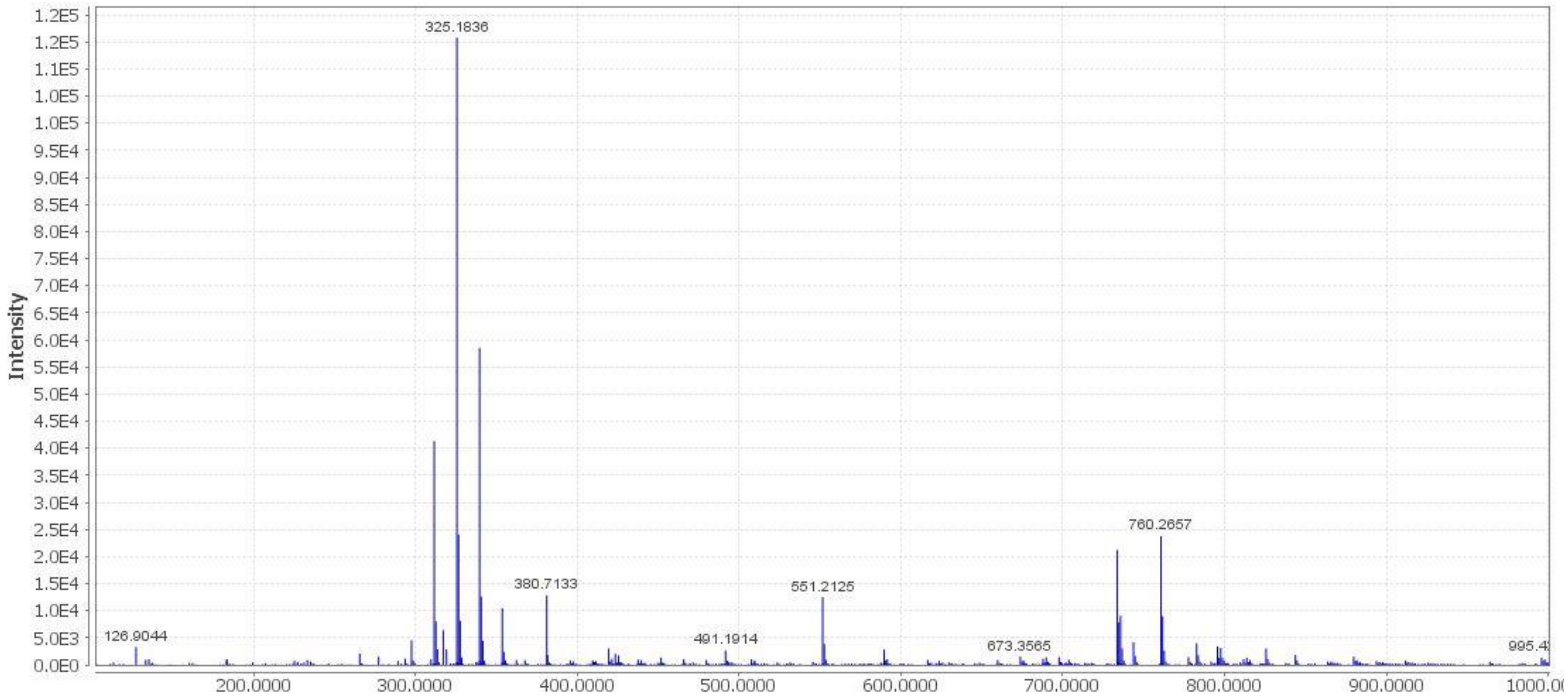

Figure 12S. MS spectra negative mode of erytrholide X

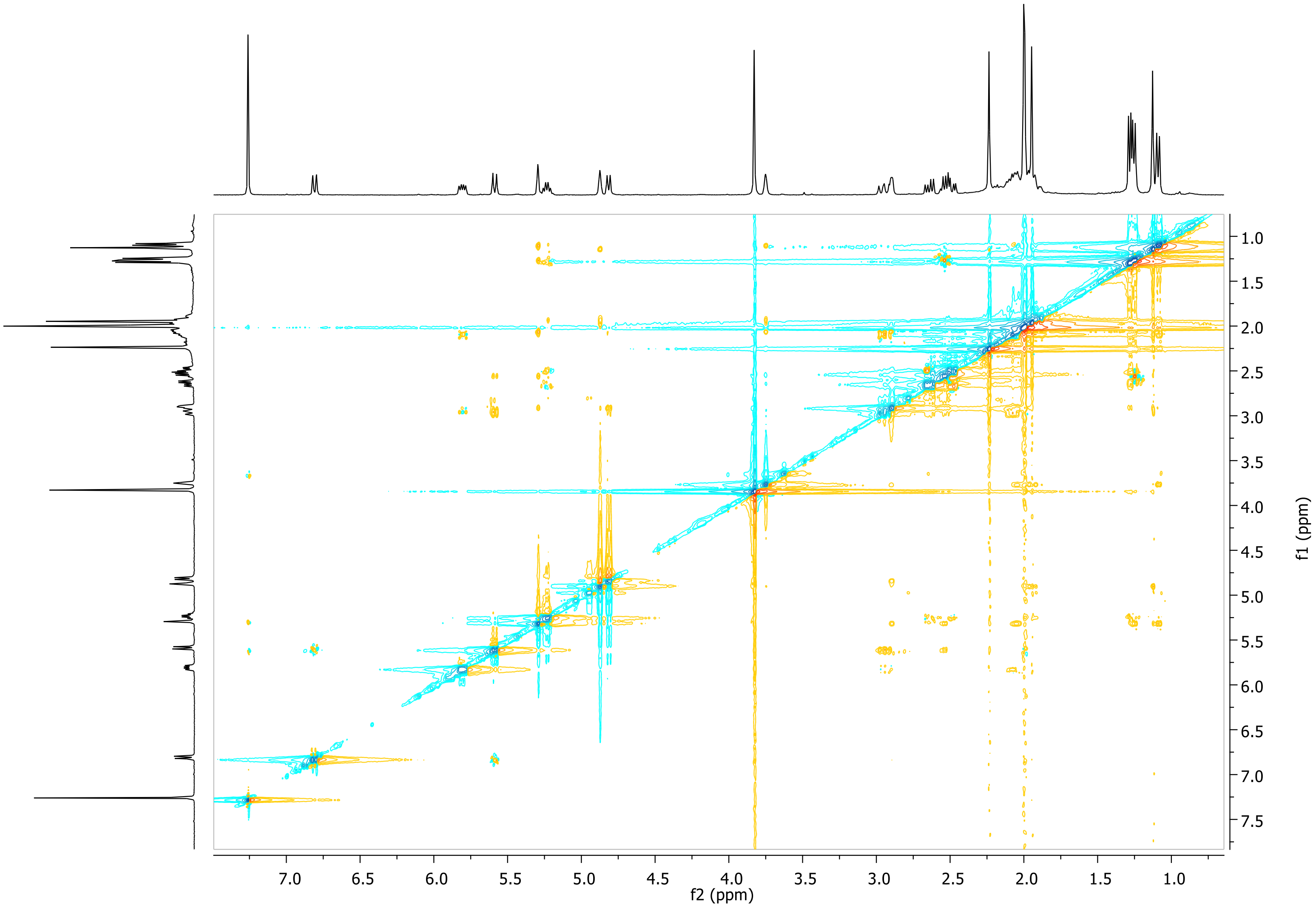

Figure 13S. NOESY spectra of erytrholide X in $CDCl_3$
